# Tofacitinib for the treatment of inflammatory bowel disease-associated arthritis: two case reports

**DOI:** 10.1186/s13256-023-03796-2

**Published:** 2023-03-01

**Authors:** Muhammad Shoaib Momen Majumder, Syed Atiqul Haq, Johannes J. Rasker

**Affiliations:** 1 Department of Rheumatology, Bangabandhu Sheikh Mujib Medical University (BSMMU), Dhaka, Bangladesh; 2 Faculty of Behavioral, Management and Social Sciences, Department Psychology, Health and Technology, University of Twente, Enschede, The Netherlands

**Keywords:** Tofacitinib, IBD-associated arthritis, Ulcerative colitis, Crohn’s disease

## Abstract

**Background:**

Musculoskeletal manifestations are common extraintestinal manifestations of inflammatory bowel disease. Tofacitinib is a Janus kinase inhibitor approved for treating rheumatoid arthritis and ulcerative colitis. There are limited data on the efficacy of tofacitinib in managing inflammatory bowel disease-associated arthritis. Here we report two patients with ulcerative colitis- and Crohn’s disease-associated arthritis successfully responding to tofacitinib.

**Cases:**

A 34-year-old Bangladeshi woman presented with lower limb polyarthritis for 7 years. Six months after the onset of polyarthritis, she developed abdominal pain with rectal bleeding. Colonoscopy and rectal biopsy findings confirmed ulcerative colitis. Ulcerative colitis associated arthritis was diagnosed. Treatment with sulfasalazine, etanercept, adalimumab, infliximab, and methotrexate gave no long-lasting remission. Methotrexate with mesalazine gave a partial response, and tofacitinib 5 mg twice per day was added. Her articular and abdominal symptoms improved within a month, and remission persisted till 24 months of follow-up, except a short-lasting mild flare at the seventh month. A 52-year-old Bangladeshi man had Crohn’s disease for 5 years. He presented with a swollen left knee and pain in other joints. Laboratory showed positive HLA-B27. He was intolerant to sulfasalazine and experienced poor response to methotrexate. Due to his inability to afford anti-tumor necrosis factor, tofacitinib was initiated. His arthritis improved within a month, and he remained in remission up to the sixth month.

**Conclusions:**

In a woman with ulcerative colitis associated arthritis, refractory to biologic therapy, both arthritis and colitis improved with tofacitinib. A patient with Crohn’s disease-associated arthritis went into remission with tofacitinib. Tofacitinib may be effective in inflammatory bowel disease-associated arthritis.

## Introduction

The most important inflammatory bowel diseases (IBDs) are ulcerative colitis (UC) and Crohn’s disease (CD). UC affects the colonic mucosa and is characterized by relapsing and remitting episodes, mainly presenting with bloody diarrhea [1]. CD can involve any part of the gastrointestinal tract, extending from the oral cavity up to the perianal area, manifested by transmural inflammation and skip areas of inflammation. Extraintestinal manifestations (EIM) are common in UC and CD, affecting 25–40% of patients with IBD. The most commonly involved organs are musculoskeletal and dermatological systems, although nearly any system can be involved [2]. The EIM can cause significant difficulty and form a challenge in the management of these patients. Arthritis occurs in 6–46% of patients with IBD, and spondylitis in 1–26% [3]. Arthritis may predate IBD symptoms in 10–30% of patients [2]. The treatment of IBD-associated arthritis consists of nonsteroidal anti-inflammatory drugs (NSAIDs), local glucocorticoid injection, sulfasalazine, methotrexate (MTX), biologics such as tumor necrosis factor inhibitors, and ustekinumab blocking interleukins 12 and 23. [4]. Though the US Food and Drug Administration (US FDA) approved tofacitinib for moderate to severe ulcerative colitis, there are few data on the efficacy of tofacitinib in IBD arthritis [5]. Here we report two cases of IBD-related arthritis (UC and CD) successfully treated with tofacitinib.

## Case 1

**Fig. 1:**
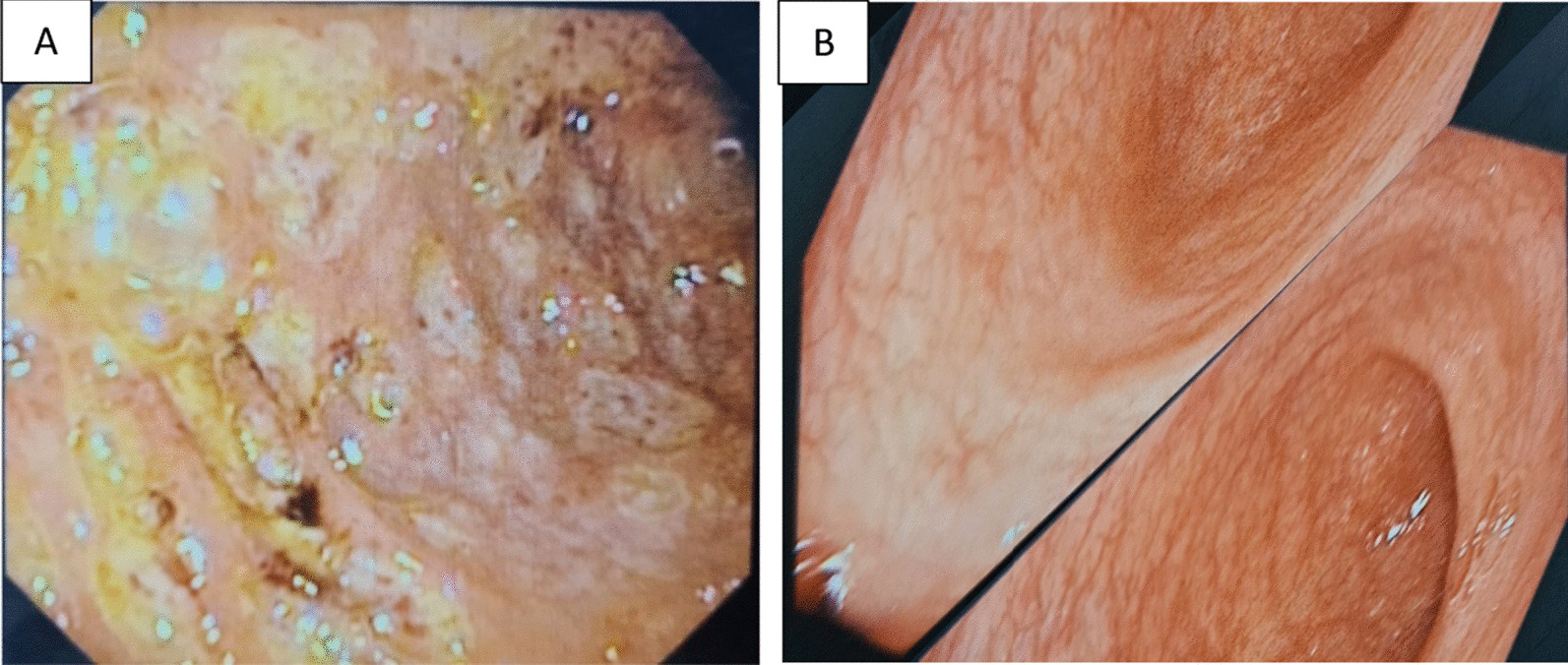
**A** Extensive ulcerative lesions before starting tofacitinib. **B** Resolution of ulcers after starting tofacitinib

**Fig. 2:**
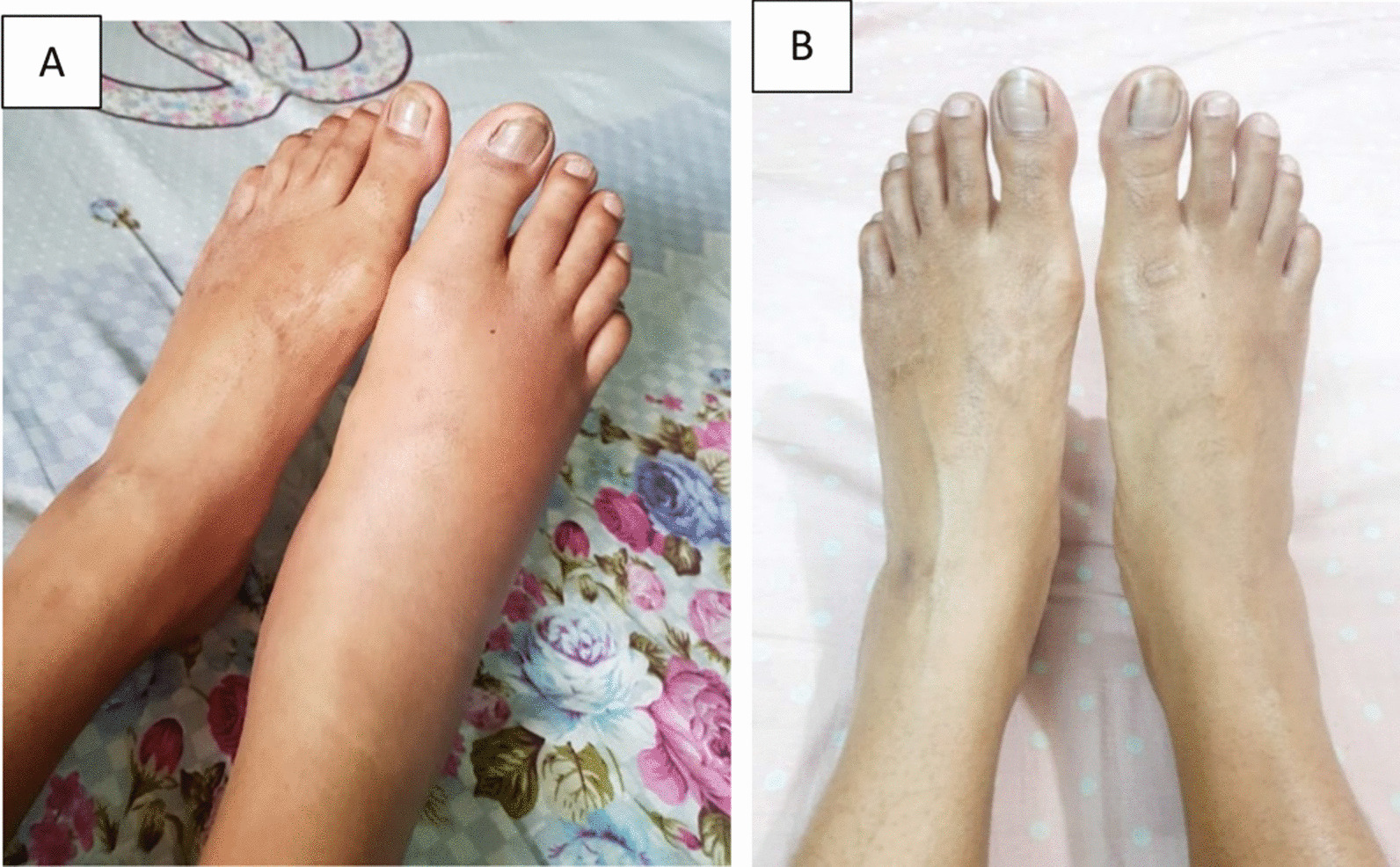
**A** Right ankle arthritis before tofacitinib. **B** Resolution of ankle arthritis with tofacitinib treatment

A 34-year-old Bangladeshi woman, normotensive, nondiabetic, and nonsmoker, developed anemia and inflammatory polyarthritis 7 years ago. The joint pain was asymmetrical, predominantly involving the large and small joints of the lower limbs. Her initial complete blood count revealed hemoglobin 9.7 g/dl, mean corpuscular volume (MCV) 72 fl (82–100 fl), mean corpuscular hemoglobin (MCH) 23 pg (27–32 pg), and ferritin 18 ng/ml (20–120 ng/ml) suggesting iron-deficient anemia, with high erythrocyte sedimentation rate (ESR) 89 mmHg and C-reactive protein (CRP) 68 mg/l (normal < 10 mg/l), and normal hepatic and renal functions. HLA-B27 and rheumatoid factor (RF), anti-CCP antibody, antinuclear antibody (ANA), and anti-dsDNA antibody were all negative. She was initially diagnosed with spondyloarthritis (peripheral) and put on sulfasalazine, on the basis of her clinical pattern. The patient responded well to sulfasalazine (the highest dose was 2.5 g per day) for 1 year. She developed abdominal discomfort, postprandial abdominal pain, loose motions, and rectal bleeding 6 months after polyarthritis. Her highest CRP rose to 109 mg/l (normal < 10 mg/l). After a flare of gastrointestinal (GI) symptoms and arthritis, she was put on etanercept 40 mg. Initially, her gastrointestinal symptoms improved, but were exacerbated at the end of 1 year with partial improvement of articular manifestations. After 16 months, she suddenly experienced severe abdominal pains with bloody diarrhea and weight loss of 5 kg within 2 months. Colonoscopy revealed marked patchy erythema with multiple erosions extending from the rectum to ascending colon and cecum (Fig. 1A). *Mycobacterium tuberculosis* (MTB) real-time polymerase chain reaction (PCR) from colonic tissue did not detect *Mycobacterium tuberculosis*, or non-tubercular mycobacteria. The colorectal biopsy showed multiple bits of chronically inflamed mucosa with focal surface ulceration covered by granulation tissue and inflammatory necrotic debris, increased cellularity of lamina propria by chronic inflammatory cells, and the presence of some crypt abscess, and ulcerative colitis (UC)-associated arthritis was diagnosed. Etanercept was stopped and switched to adalimumab 40 mg twice per month. Though her symptoms improved initially, she experienced an articular flare at the sixth month. Then she was treated with infliximab combined with methotrexate, and initially, her joint pain decreased, and she had an incomplete resolution of her abdominal symptoms. After 12 months, the articular manifestations relapsed (Fig. 2A), and treatment was stopped. As her joint pain and abdominal symptoms were resistant to sulfasalazine, etanercept, adalimumab and infliximab, and she had very active disease with ESR 77 mmHg and CRP 51.8 mg/dl, she was switched to tofacitinib 5 mg twice daily combined with MTX 20 mg per week, in the background of mesalazine 1200 mg. The patient took mesalazine for 3 months before starting tofacitinib, with partial response of her GI manifestations, and experienced mild increase in stool frequency if she stopped mesalazine. Her musculoskeletal (Fig. 2B) and abdominal symptoms responded well within a month of starting tofacitinib, and the improvement persisted for 7 months. Then her abdominal pain and bloating increased with a new development of peroneal tenosynovitis (pain, tenderness, and mild swelling over the left lateral foot). The dose of tofacitinib was increased to 15 mg daily and mesalazine to 2400 mg daily. GI and musculoskeletal symptoms improved within 1 month, and tofacitinib was reduced to 10 mg daily after 3 months. Due to her good disease control, MTX was stopped in the 20th month. The patient experienced remission of GI and musculoskeletal symptoms for 24 months with tofacitinib 10 mg daily and mesalazine 800 mg daily. The ESR became 9 mmHg and CRP 3.1 mg/l in the 24th month. A colonoscopy showed resolution of all ulcers, except for very few almost healed ulcers noted by the scar (Fig. 1B).

## Case 2

**Fig. 3:**
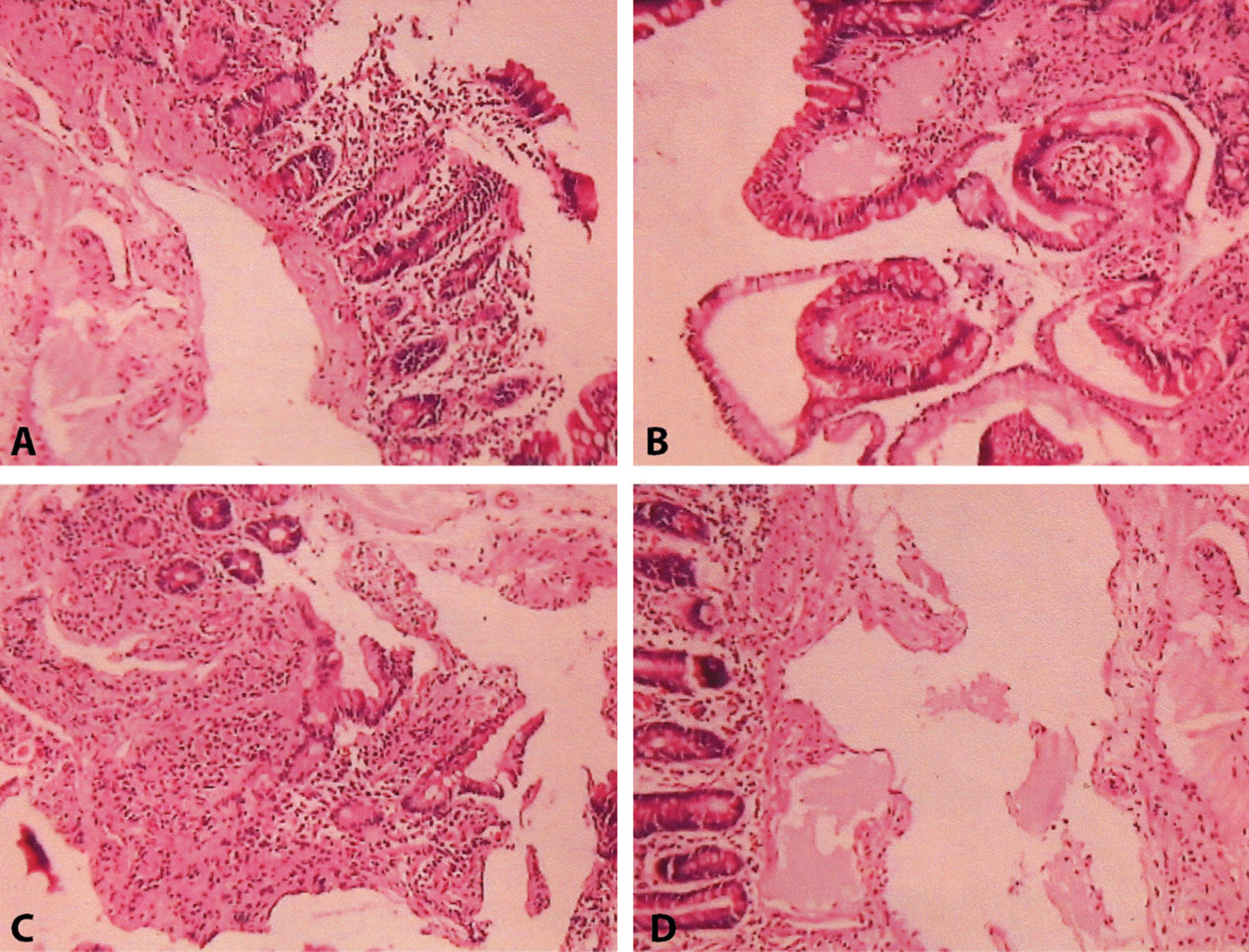
**A**–**D** Sections show jejunal mucosa with foci of surface erosion (**A**) and edema of lamina propria (**B**), along with diffuse infiltration of lymphocytes and few plasma cells (**C**), including focal intraepithelial lymphocytes (**A**, **B**). Muscularis mucosa is fibrotic (**C**). Submucosa shows dilated lymph vessels (**B**, **D**). No granuloma is seen [hematoxylin and eosin (HE), ×100]

A 52-year-old nonsmoker, hypertensive Bangladeshi male, presented with a 5 year history of Crohn’s disease. Initially, he suffered from abdominal bloating and postprandial loose motions. Enteroscopy revealed multiple aphthoid ulcers in the mid-jejunum, and a tight ileal stricture that did not allow the scope to pass further. Histopathology from the jejunal ulcer tissue confirmed the diagnosis of Crohn’s disease (Fig. 3A–D). He was reasonably well treated with prednisolone, followed by mesalazine up to 2400 mg. The patient was hospitalized 8 months ago with a high fever, increased gastrointestinal symptoms, and severe pain in the joints of the lower limbs and hands. The left knee joint was hugely swollen, and he became bedridden due to severe pain. Laboratory findings showed almost normal hemoglobin 12.8 g/dl (normal 15 ± 2 g/dl), leukocytes of 13,170/ml (normal value 4000–11,000/ml) with neutrophils 68% (normal value 55–70%), lymphocytes 28% (normal value 20–40%), and thrombocytes of 681,000/ml (*N* 150,000–400,000), ESR 49 mmHg (*N* 0–10), and CRP 105 mg/l (*N* < 5). Hepatic and renal functions and fecal calprotectin were normal. Rheumatoid factor and anti-CCP were negative and HLA-B27 positive. His blood and stool cultures revealed no organism growth with negative Dengue NS1 antigen.

Finally, Crohn’s disease-associated arthritis was diagnosed. He experienced a poor response to naproxen 1000 mg and 20 mg prednisolone daily. About 36 cc of synovial fluid was aspirated from the left knee joint, and intraarticular triamcinolone was given. We initiated sulfasalazine up to 2 g daily, resulting in partial improvement but also the development of profound anorexia. The patient experienced another articular flare at the end of 2 months. His arthritis did not improve with MTX 20 mg per week. Due to persisting arthritis and lack of funds for anti-TNF, he was initiated on tofacitinib 5 mg twice daily. After 1 month, the joint pain and swelling in the knee and other joints, and the abdominal symptoms had improved significantly. The acute phase reactants became normal at the third month of starting tofacitinib, with ESR 12 mmHg and CRP 0.32 mg/l. The improvement persisted till the last follow-up at the sixth month.

## Discussion

Articular involvement is the most common EIM of inflammatory bowel disease, involving approximately 30% of patients. The musculoskeletal manifestations are more common in Crohn’s disease than ulcerative colitis [6]. Management of IBD-associated arthritis may be difficult and may pose a challenge for the treating physicians. In the cases described above, arthritis predated ulcerative colitis in one patient, and in the other case, arthritis developed 5 years after the onset of Crohn’s disease. In the first case, the patient had no lasting effects, or was refractory to multiple conventional and biologic DMARDs. After initiation of tofacitinib, the patient has been in remission (articular and gastrointestinal) for 2 years.

Due to financial constraints and intolerance for sulfasalazine, with poor response to MTX, the second patient was advised for tofacitinib. He also responded well to tofacitinib.

IBD-associated musculoskeletal manifestations can be clinically divided into peripheral and axial involvement. The components of peripheral arthropathies are type 1 and 2 peripheral arthritis, enthesitis, dactylitis, and arthralgia. Axial arthropathy may consist of isolated sacroiliitis, inflammatory back pain, and ankylosing spondylitis [7]. The treatment of enteropathic arthritis is almost similar to other forms of spondyloarthritis, except for azathioprine. Anti-TNF therapy is recommended for those who experience a poor response to NSAIDS and conventional synthetic DMARDs (for example, sulfasalazine, methotrexate) [4].

Tofacitinib is a pan-jakinib (Janus kinase inhibitor) that inhibits JAK1/JAK2/JAK3. Tofacitinib is approved by the US FDA for treating rheumatoid arthritis, psoriatic arthritis, UC, and polyarticular course juvenile idiopathic arthritis (JIA) [8]. It is a reversible, competitive inhibitor that binds to the adenosine triphosphate (ATP) binding site of the kinase domain of JAK. Cytokines that drive immunopathology bind to receptors that activate phosphotransferases (kinases) that belong to a small family named JAKs. Tofacitinib inhibits the phosphorylation and activation of JAK, which prevents the activation of signal transducer and activator of transcription (STAT) pathway. Finally, the activation of gene transcription is inhibited, leading to decreased cytokine production and immune modulation [8, 9]. Different types of cytokines and cell proteins are responsible for the pathogenesis of IBD, including the JAK-STAT pathway. Tofacitinib downregulates the production of proinflammatory cytokines such as interleukin (IL) 2, 4, 7, 9, 15, and 21 by inhibiting the JAK-STAT pathway [10]. Anti-TNF therapy has advanced IBD treatment, including the EIMs; however, about one-third of patients fail the initial treatment, and 23–46% of patients experience secondary nonresponse (lose response over time) [11]. Tofacitinib is recommended as a remission induction and maintenance agent for moderate to severely active ulcerative colitis [12], but is not well studied for the management of EIMs of IBD. In recent times, two case reports were published where the patients responded well to both their UC and rheumatological symptoms with the combination of tofacitinib and vedolizumab [13, 14]. Although there was no significant difference in tofacitinib- and placebo-treated patients in the OCTAVE induction trial, UC-associated peripheral arthritis improved in the tofacitinib-treated group in the OCTAVE Sustain trial [15]. Tofacitinib has failed to significantly improve inducing clinical response and remission for CD in the phase II trials [16]. Another case report demonstrated improvement with tofacitinib in refractory CD-associated arthritis [17]. This case series of these patients provide us with the perception that tofacitinib may be a successful treatment option for IBD-associated arthritis.

## Conclusions

Tofacitinib may be an effective treatment option for IBD-associated arthritis and is beneficial, not only if conventional DMARD fails but also in the case where biologic treatment also becomes useless in IBD-associated arthritis. We recommend large-scale clinical trials to confirm tofacitinib as a recommended drug in managing IBD-associated arthritis.

## Data Availability

All data regarding this case have been reported in this manuscript. Please contact the corresponding author if you are interested in any further information.

## Abbreviations

IBD: Inflammatory bowel disease
UC: Ulcerative colitis
CD: Crohn’s disease
EIM: Extraintestinal manifestations
NSAID: Nonsteroidal anti-inflammatory drugs
DMARD: Disease-modifying anti-rheumatic drugs
Anti TNF: Anti-tumor necrosis factor
JAK STAT: Janus kinase and signal transducer and activator of transcription
